# A study of physician collaborations through social network and exponential random graph

**DOI:** 10.1186/1472-6963-13-234

**Published:** 2013-06-26

**Authors:** Shahadat Uddin, Liaquat Hossain, Jafar Hamra, Ashraful Alam

**Affiliations:** 1 Complex System Research Centre, The University of Sydney, Sydney NSW 2006, Australia; 2 Sydney School of Public Health, Sydney Medical School, The University of Sydney, Sydney NSW 2006, Australia

**Keywords:** Physician collaboration network, Exponential random graph, Social network analysis, Hospitalisation cost and readmission rate

## Abstract

**Background:**

Physician collaboration, which evolves among physicians during the course of providing healthcare services to hospitalised patients, has been seen crucial to effective patient outcomes in healthcare organisations and hospitals. This study aims to explore physician collaborations using measures of social network analysis (SNA) and exponential random graph (ERG) model.

**Methods:**

Based on the underlying assumption that collaborations evolve among physicians when they visit a common hospitalised patient, this study first proposes an approach to map collaboration network among physicians from the details of their visits to patients. This paper terms this network as physician collaboration network (PCN). Second, SNA measures of *degree centralisation*, *betweenness centralisation* and *density* are used to examine the impact of SNA measures on *hospitalisation cost* and *readmission rate*. As a control variable, the impact of *patient age* on the relation between network measures (i.e. *degree centralisation*, *betweenness centralisation* and *density*) and hospital outcome variables (i.e. *hospitalisation cost* and *readmission rate*) are also explored. Finally, ERG models are developed to identify micro-level structural properties of (i) *high-cost* versus *low-cost* PCN; and (ii) *high-readmission rate* versus *low-readmission rate* PCN. An electronic health insurance claim dataset of a very large Australian health insurance organisation is utilised to construct and explore PCN in this study.

**Results:**

It is revealed that the *density* of PCN is positively correlated with *hospitalisation cost* and *readmission rate*. In contrast, *betweenness centralisation* is found negatively correlated with *hospitalisation cost* and *readmission rate*. *Degree centralisation* shows a negative correlation with *readmission rate*, but does not show any correlation with *hospitalisation cost*. *Patient age* does not have any impact for the relation of SNA measures with *hospitalisation cost* and hospital *readmission rate*. The *2-star* parameter of ERG model has significant impact on *hospitalisation cost*. Furthermore, it is found that *alternative-k-star* and *alternative-k-two-path* parameters of ERG model have impact on *readmission rate*.

**Conclusions:**

Collaboration structures among physicians affect *hospitalisation cost* and hospital *readmission rate*. The implications of the findings of this study in terms of their potentiality in developing guidelines to improve the performance of collaborative environments among healthcare professionals within healthcare organisations are discussed in this paper.

## Background

Collaborations among physicians have been found very important to the effectiveness in delivering healthcare services and in producing better patient outcomes [1,2]. The structure of collaboration (i.e. the way how people communicate and collaborate with others in a collaborative environment) among the hospital staff could not be the same in different hospitals or healthcare organisations. These various structures may have different impact on healthcare outcome measures (e.g. hospitalisation expenses and patient satisfaction) in various healthcare contexts [3]. Some structures could be more conducive in terms of patient and hospital outcomes compared to others. Therefore, it is necessary to analyse different structures of collaborations among healthcare professionals and their impact on outcome variables. In this paper, measures of social network analysis (SNA) and exponential random graph (ERG) models are employed to explore physician collaborations in order to find out structural attributes of physician collaborations that are conducive to *hospitalisation cost* and *readmission rate*.

Collaboration, which is a recurring process where two or more people or organisations work together towards common goals [4], enables individuals and organisations to work together more effectively and efficiently. Collaborative relationships among individuals are highly celebrated in organisations because the synergies realised by combining multi-dimensional efforts and diverse expertise produce benefits greater than those achieved through individual effort [5]. In the context of healthcare service providers or hospitals, collaboration among different healthcare professionals is recognised as a catalyst to improved patient outcomes such as less hospital length of stay and *hospitalisation cost*[6-8], lower death rate [9] and higher satisfaction [10,11]. In healthcare settings, collaboration allows input from multiple professions (e.g. nurse and physicians), which could produce decisions leading to better patient outcomes because those decisions are based on more complete information [8].

The context of this study is the physicians’ collaborations that evolve within healthcare service providers or hospitals during the course of providing healthcare services to patients. Arguably, it can be conceptualised that physicians collaborate with each other and with other hospital staffs (e.g. nurses) in order to provide effective services to hospitalised patients. Based on the patient condition and unavailability of their colleagues, physicians might seek advices or suggestions from other physicians working in different workplaces. Because of this type of medical practice culture in healthcare service providers or hospitals, a professional collaboration network has eventually been developed over time among physicians. This study terms this network as *‘Physician Collaboration Network (PCN)’*.

The measures and methods of social network analysis (SNA) have been found useful in investigating networks (e.g. PCN) and their effects on performance [12,13]. SNA can be seen as the mapping and measuring of relationships among participating actors [14] and can provide both a visual and a mathematical analysis of network relations among actors. It plays an important role in identifying and quantifying the informal network which functions at a level beyond the formal and traditional organisational structure of actor relationships [13]. In modelling structures of PCN, this study uses the Exponential Random Graph (ERG) model which is a probabilistic model and has been utilised extensively in the social science literature to study the dynamics of network formation from underlying locally prominent micro structures such as 2-star, 3-star, triangle and so on [15]. Although most of the studies about ERG focus on building the theory of ERG models, recently researchers have applied ERG models in practice, such as, to understand whether external connections beyond the department are important to the understanding of the departmental structure of an Australian Government Organisation [16], to explore the dynamics of biological networks [17] and to examining the communication dynamics of networks under stress [18]. This study considers *hospitalisation cost* and *readmission rate* as surrogate measures for the effectiveness and efficiency of physician collaborations. There are several evidences of the use of *readmission rate* and *hospitalisation cost* as outcome measures in the healthcare literature [19-21]. Further, this study utilises *patient age* as a control variable. The use of *patient age* as a control or moderating variable has been found in several studies of the present healthcare literature [22,23].

The aim of this study is threefold. It first proposes a way to map physicians’ collaboration from their visiting information to patients. Then this study explores, by considering *patient age* as control variable, what macro-level (i.e. the complete structure of a collaboration network) SNA measures of these collaboration networks affect *hospitalisation cost* and hospital *readmission rate*. The last aim of this study is to examine what micro-level structures (i.e. small structures among few physicians in a PCN such as *2-star*) among physicians affect *hospitalisation cost* and hospital *readmission rate*. For this purpose, this study considers the only top 5 collaboration networks having *low-cost* and *low-readmission rate*, and top 5 collaboration networks having *high-cost* and *high-readmission rate*. The organisation of this paper is as follows. The rest of the 'background' section reviews the current collaboration literature in healthcare context and illustrates the way to map physicians’ collaboration from their visiting information to patients. The research methodologies (i.e. description of SNA measures, ERG models, research dataset, dependent variables and control variable) followed in this study are described in the 'methods' section. The 'results' section describes the findings of this study. Finally, the 'discussion and conclusion' section discusses the findings of this study and provides some policy recommendations for healthcare managers or administrators. This section also makes a conclusion for this paper.

### Literature review: collaboration in healthcare context

There are numerous studies in current literature exploring the effect of collaboration among healthcare professionals on patient outcomes and hospital performance. Most of these studies explore hospital performance and patient outcomes by analysing collaboration networks among different healthcare professionals such as nurse-physician collaboration [9], physician-pharmacist collaboration [24], physician-patient collaboration [25], hospital-physician collaboration [26], and inter-professional and interdisciplinary collaboration [27]. Cunningham et al. [28] have conducted an orderly review of studies of professionals’ network structures, analysing factors connected with network effectiveness and sustainability, specifically in relation to the quality of care and patient safety. The authors explore MEDLINE, CINAHL, EMBASE, Web of Science and Business Source Premier from January 1995 to December 2009. A majority of 26 studies reviewed used social network analysis to analyse structural relationships in networks: structural relationships within and between networks, health professionals and their social context, health collaborations and partnerships, and knowledge sharing networks. Essential features of networks explored were administrative and clinical exchanges, network performance, integration, stability and influence on the quality of healthcare. They have also noticed that more recent studies demonstrate that cohesive and collaborative health professional networks can promote the coordination of care and contribute to improving quality and safety of care. Structural network vulnerabilities include cliques, professional and gender homophily and over-reliance on central agencies or individuals. Efficient professional networks engage basic structural network features (e.g. bridge, broker, density, centrality, degree of separation, social capital and trust) in generating collaboratively oriented healthcare. This requires effective transmission of information and social and professional interaction within and across networks. For those using networks to improve care, recurring success factors are: understanding your network’s characteristics, attending to its functioning and investing time in facilitating its improvement. Despite this, there is no guarantee that time spent on networks will necessarily improve patient care.

Another classic study, led by Knaus and his team, identifies a significant relationship between the degree of nurse-physician collaboration and patient mortality in intensive care units [9]. They study treatment and outcome in 5030 intensive care unit patients and find that hospitals where nurse-physician collaboration is presented report a lower mortality rate compared to the predicted number of patient deaths. Conversely, hospitals that are noted for poor communication among healthcare professionals exceed their predicted number of patient deaths. In a two group quasi-experiment on 1207 general medicine patients (n = 581 in the experimental group who received care from a specially designed care management plan that facilitated higher collaboration among hospital staff and n = 626 in the control group who received the usual care), Cowan et al. [6] notice average hospital length of stay, total *hospitalisation cost*, and hospital *readmission rate* are significantly lower for patients in the experimental group than the control group (5 versus 6 days, *P* < .0001) which contributes a *‘backfill profit’* of US$1591 per patient to hospitals. There are other studies that also highlight the importance of collaboration among healthcare professionals for better patient outcomes.

Sommers et al. [29] examine the impact of an interdisciplinary and collaborative practice intervention involving a primary care physician, a nurse, and a social worker for community-dwelling seniors with chronic illnesses. They conduct a controlled cohort study of 543 patients in 18 private office practices of primary care physicians. The intervention group receives care from their primary care physician working with a registered nurse and a social worker, while the control group receives care as usual from primary care physicians. They notice that the intervention group produced better result to readmission rate and average office visits to all physicians. Moreover, the patients in the intervention group report an increase in social activities compared with the control group’s decrease. There are other studies emphasising collaboration for effective patient outcome across professional boundaries within hospitals. By analysing data collected from 105 interviews (with 40 physician, 32 case managers, 23 physician office staff, 8 administrators, and 2 case assistants), Netting and Williams [30] argue that there is a growing need to collaborate and communicate across professional lines rather than make assumptions about who can do what for better patient outcomes, professional satisfaction, and hospital performance.

Like these studies, most of the collaboration studies of contemporary healthcare literature advocate for the effective and efficient collaboration among healthcare professionals for better patient care. Proper collaborations among hospital staff positively drive both total *hospitalisation cost* and hospital *readmission rate*. There are many other studies in healthcare context that analyse networked collaboration among healthcare specialists to explore different aspects of professional behaviour and quality patient care, such as, to evaluate the effects of GP network organisation on their prescribing behaviour [31] and to develop a selection criteria of group members in order to improve the effectiveness of team-based approach to patient care [32]. However, none of these studies, to our knowledge, provides any guidelines: (i) about the network structure of effective collaboration; (ii) what type of collaboration structure is more conducive compared to others; and (iii) on how individual healthcare professional should develop relations with others over time in a collaborative environment for better performance. This study considers only the physician collaboration and addresses all of these three issues by exploring PCNs using measures of social network analysis (SNA) and exponential random graph (ERG) models.

### Physician collaboration network (PCN)

Collaboration in healthcare is defined as healthcare professionals accepting complementary roles and jointly working together, sharing accountability for problem-solving and making decisions to develop and implement plans for patient care [33,34]. Collaboration among physicians, nurses and other healthcare professionals increases team members’ perception of each other’s type of knowledge and skills, leading to continued improvement in decision-making [35]. It can take place in both face-to-face interactions and electronically via fast-paced encounters such as e-mail. In whatever location or form, collaboration includes an exchange of beliefs and ideas that acknowledges the perspectives of all collaborators, whether or not agreement is accomplished in the interaction [36]. To minimise misunderstandings, it is also essential to define what is not implied by the term ‘*collaboration*’. It does not imply supervision, nor is it simply a one-way or two-way information exchange. Efficient professional collaborative relationships require mutual respect [37]. They also call for trust and diligence. In complex and sophisticated healthcare systems, collaboration is generally challenging. Collaboration may seem idealistic and perhaps even non-realistic. However, Kramer and Schmalenberg [37] state that collaborative partnerships are worth the effort because they result in improved effects for patients as well as individual development for collaborators.

Collaboration between physicians has been poorly investigated; the overwhelming focus of research on physicians has been on their collaboration with patients [38]. Interestingly, research on collaboration between physicians has focused on the discussion of medical mistakes, collegial control and other negatively recognised aspects of medical care [39-42]. One area that has been seen critical is the culture of medicine and the socialisation of medical students, interns, and residents into that culture by physicians [43,44]. Atkinson [38] argues that “*biomedical knowledge is socially produced and culturally specific . . . [and] dependent upon certain fundamental features of medical culture, which is itself produced and reproduced through processes of socialisation*” (p. 46). His study of haematologists’ consultations with physicians of other specialties demonstrates the method of generating medical knowledge through collaboration among physicians. According to Atkinson, physicians’ communication is not the way to the accomplishment of medical work; the communication is the work.

In this study, it is assumed that collaborations among physicians emerge when they visit common hospitalised patients. It is a standard professional practice around the world that when physicians visit patients, they give advice or suggestions to patients based on their health condition and previous medication history deposited in the patient *log book*. All previous advice or suggestions by any physician to a patient have been taken into consideration during any subsequent physician visit to that patient. This kind of practice culture in healthcare organisations or hospitals enables us to map and, eventually, to model PCNs.

**Figure 1:**
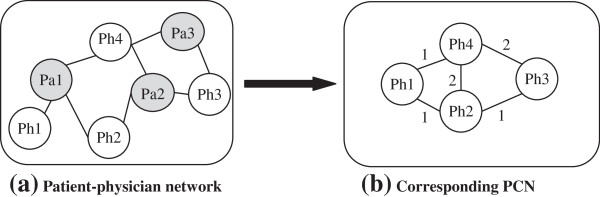
**Conceptualisation of the collaboration network among physicians.** (**a**) *Patient-physician* network, and (**b**) Corresponding PCN (*Pa* stands for patient, and *Ph* stands for physician.

When physicians visit common patients within the same hospital or healthcare organisation PCN emerges among them. Figure 1 illustrates an example of such a PCN construction. In a hospital (say *H1*), patient *Pa1* is visited by *Ph1*, *Ph2* and *Ph4* physicians, and patient *Pa2* is visited by *Ph2*, *Ph3* and *Ph4* physicians, and physician *Ph3* and *Ph4* visit patient *Pa3*. This is depicted in the *patient-physician* network in Figure 1(a). The corresponding PCN for this *patient-physician* network is demonstrated in Figure 1(b). In this PCN, there are network connections with weight 1 between *Ph1* and *Ph2*, between *Ph1* and *Ph4*, and between *Ph2* and *Ph3* because they visit only one common patient. The weight of the links between *Ph2* and *Ph4*, and between *Ph3* and *Ph4* are 2 as they have two common patients.

## Methods

In explaining different SNA measures and ERG model, this study uses the terms actor(s) and node(s) interchangeably. Similarly, the words link(s) and tie(s) are exchangeable in this paper.

### Measures of social network analysis (SNA)

This study utilises SNA measures of *degree centralisation*, *betweenness centralisation*, and *network density*. The selection of these three measures is guided by two network theories: (i) Bavelas’ Centralisation Theory [45]; and (ii) Freeman’s Centrality Theory [46]. These two theories can explain structural influences of collaboration and communication networks on the group performance.

#### Degree centralisation and betweenness centralisation

Before explaining *degree centralisation* and *betweenness centralisation*, it is required to define *degree centrality* and betweenness *centrality*. Centralisation is a network-level measure whereas centrality is a node-level measure; thus, the later one needs to be explained first before describing the former one. Centrality is an important concept in studying networks. In conceptual terms, centrality measures how central an individual is positioned in a network. *Degree centrality* is one of basic measures of network centrality. For an actor, it is the proportion of nodes that are adjacent to that actor in a network. It highlights the node with the most links to other actors in a network, and can be defined by the following equation for the actor *i* in a network having *N* actors [13]:

(1) $$C_{D}^{'}\left(n_{i}\right)=\frac{d\left(n_{i}\right)}{N-1}$$

Where, the subscript *D* for *degree* and *d*(*n*_*i*_) indicates the number of actors with whom actor *i* is connected. The maximum value for $C_{D}^{'}\left(n_{i}\right)$ is 1 when actor *i* is linked with all other actors in the network. For an isolate actor, its value is 0.

*Betweenness centrality* views an actor as being in a favoured position to the extent that the actor falls on the shortest paths between other pairs of actors in the network. That is, actors that occur on many shortest paths between the other pair of nodes have higher *betweenness centrality* than those they do not [46]. The *betweenness centrality* for an actor *n*_*i*_ (i.e. C_B_(n_i_)) can be represented by the following equation [13]:

(2) $$C_{B}^{'}\left(n_{i}\right)=\frac{\sum_{j<k}\frac{g_{\mathrm{jk}}\left(n_{i}\right)}{g_{\mathrm{jk}}}}{\left[\left(N-1\right)\left(N-2\right)\right]/2}$$

Where*, i ≠ j ≠ k*; *g*_*jk*_(*n*_*i*_) represents the number of the shortest paths linking the two actors that contain actor *i*; and *g*_*jk*_ is the number of the shortest paths linking actor *j* and *k*. For the central actor of a start, $C_{B}^{'}\left(n_{i}\right)$ will take its highest value of 1; however, for any peripheral actor of a star $C_{B}^{'}\left(n_{i}\right)$ will take its minimum value of 0.

A centralisation measure quantifies the range or variability of individual actor indices. The set of *degree centralities*, which represents the collection of *degree* indices of *N* actors in a network, can be summarised by the following equation to measure network *degree centralisation*[47]:

(3) $$C_{D}=\frac{\sum_{i=1}^{N}\left[C_{D}\left(n^{*}\right)-C_{D}\left(n_{i}\right)\right]}{\left[\left(N-1\right)\left(N-2\right)\right]}$$

Where, {*C*_*D*_(*n*_*i*_)} are the *degree* indices of *N* actors and *C*_*D*_(n*) is the largest observed value in the *degree* indices. For a network, *degree centralisation* (i.e. the index *C*_*D*_) reaches its maximum value of 1 when one actor chooses all other *(N-1)* actors and the other actors interact only with this one (i.e. the situation in a *star* graph). This index (i.e. *C*_*D*_) attains its minimum value of 0 when all *degrees* are equal (i.e. the situation in a *circle* graph). Thus, *C*_*D*_ indicates varying amounts of centralisation of *degree* compared to both *star* and *circle* graph.

Similarly, the set of *betweenness centralities*, which represents the collection of *betweenness* indices of *N* actors in a network, can be summarised by the following equation to measure network *betweenness centralisation*[47]:

(4) $$C_{B}=\frac{\sum_{i=1}^{N}\left[C_{B}^{'}\left(n^{*}\right)-C_{B}^{'}\left(n_{i}\right)\right]}{\left(N-1\right)}$$

Where, $\left\{C_{B}^{'}\left(n_{i}\right)\right\}$ are the *betweenness* indices of *N* actors and $C_{B}^{'}\left(n^{*}\right)$ is the largest observed value in the *betweenness* indices. Freeman [46] demonstrates that *betweenness centralisation* reaches its maximum value of 1 for the *star* graph. Its minimum value of 0 occurs when all actors have exactly the same *betweenness* index.

#### Network density

The *density* of a network represents the proportion of existing ties (or, links) relative to the maximum number of possible ties among all actors of that network [13]. The *density* value for a network is 1 only when all the actors of that network are connected with each other. On the other hand, for a completely sparse network, the density value is 0, which indicates there is no link exists between any two actors of that network. For an undirected network of size *N* (i.e. have *N* actors), theoretically there are [*N* * (*N* − 1)]/2 (i.e. C2N) possible links among its *N* actors. If there are *N*_*t*_ links among its *N* actors in that network, then, mathematically, *density* can be defined as [13]:

(5) $$\mathrm{Density}=\frac{2*N_{t}}{N*\left(N-1\right)}$$

### Exponential random graph (ERG) models

ERG model can effectively identify structural properties in social networks [48]. This theory-driven modelling approach also allows to test the significance of structural parameters in the process of the formation of a given network [18,49]. For instance, a given cost effective PCN may be explored using ERG model to examine what micro structures play a statistically significant role in the development process of this PCN. It simplifies a complex structure down to a combination of basic parameters. The advantage of this approach is that it is very general and scalable as the architecture of the graph is represented by locally determined explanatory variables, and the choice of explanatory variables is quite flexible and can be easily revised. The disadvantage of this approach is the difficulty in estimating the execution time. The reason for that is that ERG models are based on simulation and execution time for simulation is always unpredictable [15]. Another disadvantage of ERG models is the complex interpretation when multiple parameters are considered and the difficulty to get convergence sometimes [15].

This paper follows the notation and terminology described in Robins et al. [50]. For each pair *i* and *j* of a set of *N* actors, *X*_*ij*_ is a network tie variable with *X*_*ij*_*= 1* if there is a network tie from *i* to *j*, and *X*_*ij*_ = 0 otherwise. This paper specifies *x*_*ij*_ as the observed value of *X*_*ij*_ with *X* the matrix of all variables and *x* the matrix of observed ties of the network. *X* may be directed or non-directed. A configuration is a set of nodes and a subset of ties among them. For example, an *edge* is a subset of two nodes in which one node is connected by a tie to other, and a *3-star* is a subset of four nodes in which one node is connected by a tie to each of the other three nodes. Similarly, *n-star* is a subset of *n* nodes in which one node is connected by a tie to each of the other *(n-1)* nodes. Configurations are defined hierarchically, so that a triangle also includes three *2-stars*. The general form of the class of (homogeneous) ERG models is as follows [50]:

(6) $$\mathrm{Pr}\left(X=x\right)=\frac{1}{k}\exp\left\{\sum_{A}\eta_{A}g_{A}\left(x\right)\right\}$$

Where, (i) the summation is over configuration types *A*; different sets of configuration types represent different models (e.g. dyadic independence or Markov random graph); (ii) *η*_*A*_ is the parameter corresponding to a configuration of type *A*; (iii) *g*_*A*_*(x)* is the network statistic corresponding to configuration *A* (for homogeneous Markov graph models this is the number of configurations of type *A* observed in the network: for example, the number of triangles); and (iv) *κ* is a normalising quantity to ensure that Eq. (6) is a proper probability distribution.

A commonly used sub-class of ERG models is the Markov random graph in which a possible tie from *i* to *j* is assumed conditionally dependent only on other possible ties involving *i* and/or *j*[51]. This sub-class of ERG model is also known as the *low-order* model. An example of a Markov random graph model for non-directed networks, with *edge* (or, *density*), *2-star*, *3-star* and *triangle* parameters, is given below [52]:

(7) $$\mathrm{Pr}\left(X=x\right)=\frac{1}{k}\;\exp\;\left\{\mathrm{\theta L}\left(x\right)+\sigma_{2}S_{2}\left(x\right)+\sigma_{3}S_{3}\left(x\right)+\mathrm{\tau T}\left(x\right)\right\}$$

**Figure 2:**
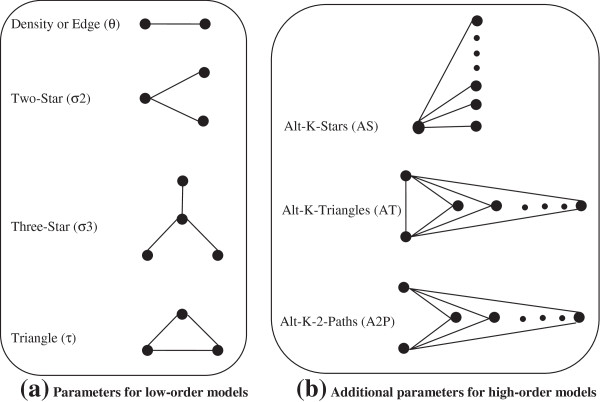
**Configurations and parameters for exponential random graph models [**[50]**].**

In Eq. (7), *θ* is the *density or edge* parameter and *L(x)* refers to the number of edges in the graph *x*; *σ*_*k*_ and *S*_*k*_*(x)* refer to the parameter associated with *k-star* effects and the number of *k-stars* in *x*; while *τ* and *T(x)* refer to the parameter for triangles and the number of triangles, respectively. For a given observed network *x*, parameter estimates indicate the strength of effects in the data. For instance, a large and positive estimate for σ_2_ suggests that, given the observed number of edges and stars, networks with more *2-stars* are more likely. The configurations and parameters of Markov random graph model (i.e. low-order model) is shown on Figure 2a. These parameters relate to some well-known structural regularity in the network literature and represent structural tendencies in the network (e.g. mutuality and transitivity). They were chosen because they are conceptualised as forces which drive the formation of the network itself. For example, *transitivity* is conceptualised as a force which drives the formation of the network itself (the friends of our friends are more likely to be our friends). Snijders et al. [15] later propose three new configurations (i.e. *alternating k-stars, alternating k-triangles* and *alternating independent two-paths*) that can be included in specifications for ERG models. They define a new sub-class of Markov random graph model (i.e. high-order model), which considers parameters of both Figure 2a,b. This study utilises both high- and low-order ERG models for modelling PCNs.

There are two methods commonly used in the statistics and social network communities to estimate the maximum likelihood fit to ERG models: Markov chain Monte Carlo maximum likelihood estimation and maximum pseudo-likelihood estimation. They can also be used for network simulation. To date, the most common form of estimation for Markov random graph models is the maximum pseudo likelihood [53]. The properties of the pseudo-likelihood estimator are not well understood and the pseudo-likelihood estimates can at best be thought of as approximate. Also, it is not clear from existing research as to when pseudo-likelihood estimates may be acceptable. Therefore, Monte Carlo Markov chain maximum (MCMC) likelihood estimation, when available, is the preferred estimation procedure. This study also uses this estimator. That means this study utilises Markov random sub-class of ERG models for modelling cross-sectional PCNs and maximum Monte Carlo Markov chain maximum (MCMC) estimator for estimation purpose.

### Research dataset

This research utilises health insurance claim dataset to explore physician collaborations using measures of SNA and ERG models. This dataset is provided by a non-profit health insurance organisation (i.e. Hospital Contribution Fund, HCF), which is the third largest health insurance organisation in Australia. It includes members’ claim data from January 2005 to February 2009. This dataset contains mainly three different categories of claim information: (i) ancillary claim (lodged by hospital); (ii) medical claim (lodged by doctor or physicians); and (iii) hospital claim (lodged by hospital). *Ancillary claims* are auxiliary claims for medical services such as dental, optical, physiotherapy, dietician, and pharmaceutical. All claims lodged by specialist physicians, except of the ancillary type, are *medical claims*. The claims for the services provided to hospitalised patients in private or public hospitals that are approved by the Department of Health, Australia are considered as *hospital claims*. In general, patients have *medical claims*, *hospital claims*, and very few *ancillary claims* for their admissions to hospitals. This study uses the claim information to construct PCN for a particular type of hospitalised patients (e.g. knee surgery patient).

As people have hospital admissions for a wide range of illness and patients with a particular disease need to be seen by particular specialist physicians, different types of PCNs (e.g. a PCN for knee surgery patients and a PCN for heart surgery patients) are being evolved inside a hospital for hospitalised patients suffering from different types of diseases. For research analysis purpose, this study considers PCNs only for total hip replacement (THR) patients from 85 different hospitals where at least 5 THR patients get admitted during the data collection period. So, 85 PCNs evolved within these 85 hospitals. In these hospitals, 2229 patients get admitted during our data collection period. These patients lodged in total 1383 ancillary claims, 65871 medical claims, and 23369 hospital claims. The basic statistics of these 85 PCNs is given in Table 1 (last column).

**Table 1:** Summary statistics of 5 low-cost, 5 high-cost, 5 low-readmission, 5-high readmission rate and the total 85 PCNs

**Item**	**Hospitalisation cost**		**Readmission rate**		**All PCNs (85)**
	***Low (5)***	***High (5)***	***Low (5)***	***High (5)***	
Average cost per patient ($AUD)	16582	29949	18931	26400	24010
Average readmission rate (%)	7.47	10.98	0.00	21.94	11.64
Average number of patient per PCN	61.8	85.8	19.2	30.8	26.22
Average number of doctors’ visit per patient	17.01	34.79	29.55	26.66	26.03
Average patients’ age (year)	54.08	67.87	73.06	65.81	68.78
Average hospital length of stay (day) per patient	4.87	13.52	8.96	12.21	10.51

To explore physician collaborations using SNA measures, these 85 PCNs are used in this research. However, for ERG modelling, this study utilises 20 PCNs. In particular, this study considers 5 most expensive PCNs (termed as *high-cost* PCNs) and 5 least expensive PCNs (termed as *low-cost* PCNs) to explore, using ERG models, how micro-level network structures varied for PCNs having different total hospitalisation cost. For modelling PCN in terms of *readmission rate*, this study considers 5 PCNs that have the highest *readmission rate* (termed as *high-readmission* PCNs) and compare their structures with 5 PCNs which have the lowest *readmission rate* (termed as *low-readmission* PCNs). The basic statistics of these 20 PCNs is given in Table 1 (the first 5 columns).

### Dependent variable: Hospitalisation cost and readmission rate

#### Hospitalisation cost

In calculating *hospitalisation cost*, this study considers all payments made by the health insurance organisation for each THR patient to the health service providers, regardless of how much that patient pays in return to the health insurance organisation (which depends on the health insurance policy type and percentage of coverage amount).

#### Readmission rate

For any PCN, *readmission rate* represents the ratio of patients (in percentage) who have hospital admissions more than once for their THR surgeries. That means,

(8) $$\begin{array}{l} \mathrm{Readmission}\;\mathrm{Rate}\left(\mathrm{PCN}\right)\\ =\frac{\mathrm{No}.\mathrm{of}\;\mathrm{THR}\;\mathrm{patient}\left(s\right)\;\mathrm{who}\;\mathrm{admitted}\;\mathrm{more}\;\mathrm{than}\;\mathrm{once}\;}{\mathrm{Total}\;\mathrm{admitted}\;\mathrm{THR}\;\mathrm{patients}}\times 100\% \end{array}$$

### Control variable: patient age

For each PCN, the average age for all patients is calculated. This average age is considered as control variable to investigate whether the *patient age* has any impact for the relation of SNA measures of PCN with *hospitalisation cost* and hospital *readmission rate*.

## Results

This section reports the results of this study.

### Mapping physician collaboration network (PCN) from insurance claim dataset

**Figure 3:**
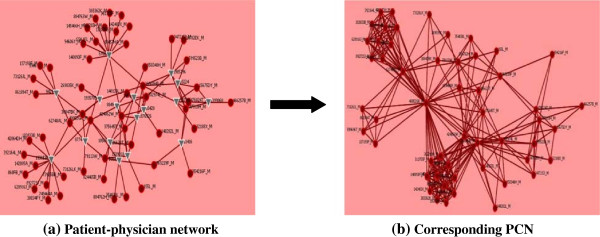
**Construction of PCN from research dataset.** The red circle represents physician and the gray triangle represents patient.

From the *medical claim* details of HCF dataset, the number of physicians visit a particular hospitalised patient during her or his hospitalisation period can be revealed because physicians make a *medical claim* to HCF for every single visit to hospitalised patients. Based on this information and by applying the PCN development approach (as illustrated in Figure 1) and process (described in section 3), the structure of PCN of each hospital for THR patients has been constructed. An example of the construction of PCN structure from the research dataset is given in Figure 3. Organisation Risk Analyser (ORA), which is a meta-network assessment and analysis tool [54], is utilised to construct such PCNs.

### Social network analysis (SNA) measure and physician collaboration network (PCN)

SNA measures and their impact on the *hospitalisation cost* and *readmission rate* are illustrated in Table 2. This table also shows the descriptive statistics (i.e. mean and standard deviation) of each variable. Although *degree centralisation* does not show a correlation with *hospitalisation cost* (rho = 0.112, p>0.05 at 2-tailed), it shows negative correlation with *readmission rate* (rho = − 0.373, p<0.01 at 2-tailed). An increase in *degree centralisation* produces a downturn for *readmission rate*. *Density* of PCN is positively correlated with both *hospitalisation cost* (rho = 0.282, p<0.01 at 2-tailed) and *readmission rate* (rho = 0.358, p<0.01 at 2-tailed). Both *hospitalisation cost* and *readmission rate* of a hospital change proportionally with the change in the *density* of the PCN of that hospital. On the other hand, the correlation coefficient values of Table 2 reveals that *betweenness centralisation* of PCN is negatively correlated with both *hospitalisation cost* (rho = −0.264, p<0.05 at 2-tailed) and *readmission rate* (rho = −0.283, p<0.01 at 2-tailed). As it is always expected to have low *hospitalisation cost* and *readmission rate*, this result indicates that low *betweenness centralisation* is not conducive for healthcare service providers or hospitals. We also develop simple linear regression models for each of hospital outcome variables (i.e. *hospitalisation cost* and *readmission rate*) and PCN estimates (i.e. *degree centralisation*, *betweenness centralisation * and *density*). These models, as described in Table 3, allow checking relative influence and independence in the associations of independent network variables and dependent hospital outcome variables. All models, except the first model (i.e. considering *degree centralisation* and *hospitalisation cost*), show statistically significant output.

**Table 2:** Descriptive statistics (M indicates mean and STD indicates standard deviation) and pair wise correlation coefficient values for all variables (i.e. SNA variables, hospitalisation cost and readmission rate) used in this study

	**[1]**	**[2]**	**[3]**	**[4]**	**[5]**
[1] **Degree centralisation**					
(M = 0.75 and STD = 0.13)					
[2] **Betweenness centralisation**	−0.062				
(M = 0.25 and STD = 0.14)					
[3] **Network density**	0.045	−0.046			
(M = 0.27 and STD = 0.11)					
[4] **Hospitalisation cost**	0.112	−0.264^*^	0.282^**^		
(M = 24009.9 and STD = 6783.3)					
[5] **Readmission rate**	−0.373^**^	−0.283^**^	0.358^*^	0.098	
(M = 11.64 and STD = 8.48)					

^**^. Correlation is significant at the 0.01 level (2-tailed).

^*^. Correlation is significant at the 0.05 level (2-tailed).

**Table 3:** **Linear regression models between each of network attributes (i.e. *degree centralisation*, *betweenness centralisation *and *density*) of PCN and hospital performance measures (i.e. *hospitalisation cost *and *readmission rate*)**

**Model**	**Dependent variable**	**Independent variable**	**R**^**2 **^**value**	**β**	**Constant**	**Significance**
1	Hospitalisation cost	Degree centralisation	0.012	5906.42	19545.39	0.309
2	Readmission rate	Degree centralisation	0.139	−37.87	45.48	0.003
3	Hospitalisation cost	Betweenness centralisation	0.107	−12384.79	27101.96	0.015
4	Readmission rate	Betweenness centralisation	0.112	−25.18	18.19	0.010
5	Hospitalisation cost	Density	0.118	17310.51	19635.69	0.009
6	Readmission rate	Density	0.196	49.08	3.427	0.000

The effect of *patient age* as control (or moderating) variable is summarised in Table 4. We develop regression models by considering each of the network measures and its product with *patient age*. To show controlling effect, the product of network measure and *patient age* must show significant association with hospital outcome variables in these regression models [55]. Out of these six models, this product shows a significant association in only two cases (i.e. the second and the third models of Table 4). That means *patient age* moderates only the relations of *betweenness centralisation* and *density* of the PCN with *hospitalisation cost*. In most cases (i.e. 4 out of 6), *patient age* does not moderate the relation between PCN attributes and hospital outcome measures. This can be explained by the fact that we do consider average age of all patients in calculating *patient age* for a PCN. On the other hand, studies of present healthcare literature consider *patient age* at the individual level, not at the aggregate level as like this study.

**Table 4:** **Linear regression models for checking controlling effect of *patient age *on the relation between each of network attributes (i.e. *degree centralisation*, *betweenness centralisation *and *density*) of PCN and hospital performance measures (i.e. *hospitalisation cost *and *readmission rate*)**

**Model**	**Dependent variable**	**R**^**2 **^**value**	**Constant**	**Independent variable**	**β**	**Significance**
1	Hospitalisation cost	0.102	20016.04	Degree centralisation	-26621.75	0.084
				Degree centralisation * Age	463.16	0.102
2	Hospitalisation cost	0.227	27106.44	Betweenness centralisation	-102698.68	0.000
				Betweenness centralisation * Age	1318.36	0.000
3	Hospitalisation cost	0.188	19216.11	Density	-60258.64	0.016
				Density * Age	1155.44	0.001
4	Readmission rate	0.113	45.82	Degree centralisation	-50.37	0.078
				Degree centralisation * Age	0.18	0.622
5	Readmission rate	0.094	18.17	Betweenness centralisation	-75.92	0.110
				Betweenness centralisation * Age	0.745	0.273
6	Readmission rate	0.081	1.318	Density	20.08	0.677
				Density * Age	0.314	0.651

### Exponential random graph (ERG) model and physician collaboration network (PCN)

Pnet ^a^[1] has been used in this study to fit ERG models with different types of PCNs (i.e. *low-cost* versus *high-cost* and *low-readmission* versus *high-readmission*). After following several iterative processes, the model (i.e. *2-star*, *3-star*, *alternating-k-stars*, *alternating- k-triangles* and *alternating-k-two-paths* model) had been found to fit with PCNs. The results for this model are shown in Table 5. The weights of different micro-structures (e.g. *2-star* and *3-star*) of this model can be tested using *t-value* (also known as *t-statistics*), which is defined by dividing the *estimate* by its *standard error*. Thus, the *t*-*value* measures how many *standard errors* the *estimate* is away from zero. Generally, any *t*-*value* greater than +2 or less than −2 (i.e. absolute *t-value* is greater than 2) is acceptable. The higher the *t*-*value*, the greater the confidence is shown by the parameter under consideration as a predictor. Low *t-value* is the indication of a low reliability of the predictive power of that parameter [56]. To compare t*-values* of different networks, researchers utilise the *t-test* method [49].

**Table 5:** **The results from high-order model (i.e. *2-star*, *3-star*, *alternating-k-stars*, *alternating-k-triangles*, *alternating-k-two-paths *model)**

**Effects**	**Estimates**	**stderr**	**Est./stderr**	**Estimates**	**stderr**	**Est./stderr**	**Estimates**	**stderr**	**Est./stderr**	**Estimates**	**stderr**	**Est./stderr**	**Estimates**	**stderr**	**Est./stderr**
	**Low cost**														
		**N1**			**N2**			**N3**			**N4**			**N5**	
2-star	0.04	0.01	6.25	0.12	0.04	2.66	−0.04	0.04	−0.97	0.03	0.01	2.02	−0.02	0.02	−0.84
3-star	0.00	0.00	0.24	0.00	0.00	0.86	0.01	0.00	2.55	0.00	0.00	−0.41	0.00	0.00	3.03
AS	−1.95	0.42	−4.60	−2.39	1.24	−1.92	−1.24	0.61	−2.01	−1.36	0.38	−3.58	−3.55	1.23	−2.89
AT	1.28	0.10	12.64	−0.05	0.07	−0.73	1.37	0.19	7.04	1.64	0.14	11.54	1.43	0.11	12.68
A2P	−0.05	0.01	−5.70	−0.17	0.03	−6.02	−0.17	0.02	−7.98	−0.02	0.02	−0.85	−0.14	0.01	−15.40
	**High cost**														
		**N1**			**N2**			**N3**			**N4**			**N5**	
2-star	0.04	0.00	19.35	0.03	0.00	13.71	0.02	0.00	9.53	−0.04	0.03	−1.34	0.08	0.01	7.19
3-star	0.00	0.00	−3.84	0.00	0.00	−1.74	0.00	0.00	−1.10	0.00	0.00	2.58	0.00	0.00	−0.77
AS	−5.45	1.03	−5.28	−5.05	1.14	−4.44	−3.30	0.55	−5.96	−9.60	8.41	−1.14	−3.68	1.30	−2.83
AT	0.76	0.06	11.74	1.06	0.08	12.72	1.72	0.08	20.66	−0.43	0.06	−6.59	0.33	0.09	3.51
A2P	−0.05	0.00	−14.60	−0.06	0.00	−13.60	−0.04	0.00	−7.76	−0.23	0.02	−11.99	−0.07	0.01	−5.40
	**Low readmission rate**														
		**N1**			**N2**			**N3**			**N4**			**N5**	
2-star	−0.81	1.78	−0.45	0.05	0.03	1.76	0.01	0.06	0.21	0.03	0.02	1.41	0.02	0.09	0.16
3-star	0.08	0.42	0.18	0.00	0.00	1.75	0.01	0.00	1.89	0.00	0.00	1.40	0.01	0.01	1.06
AS	0.15	2.98	0.05	−1.76	0.77	−2.28	−5.05	1.94	−2.60	−3.21	0.89	−3.61	−0.19	0.98	−0.20
AT	1.31	0.33	3.93	0.68	0.12	5.49	0.70	0.14	4.94	1.71	0.19	8.79	−0.02	0.11	−0.20
A2P	0.17	0.36	0.46	−0.11	0.03	−4.05	−0.24	0.02	−13.12	−0.11	0.01	−9.88	−0.16	0.06	−2.76
	**High readmission rate**														
		**N1**			**N2**			**N3**			**N4**			**N5**	
2-star	0.02	0.01	0.05	0.06	0.01	−0.03	−0.02	0.01	0.02	0.05	0.03	0.04	0.30	0.26	−0.17
3-star	0.00	0.00	0.07	0.00	0.00	0.05	0.00	0.00	0.08	0.00	0.00	0.04	−0.02	0.02	−0.17
AS	−5.23	1.29	0.04	−8.65	1.25	−0.02	−6.65	2.47	0.05	−7.54	2.95	0.01	−26.01	11.73	−0.34
AT	0.48	0.11	−0.02	0.97	0.10	0.01	0.46	0.12	−0.01	0.97	0.15	0.03	−0.07	0.07	−0.34
A2P	−0.15	0.01	0.05	−0.11	0.00	0.05	−0.18	0.01	0.09	−0.11	0.02	−0.04	−0.33	0.01	0.09

The parameter interpretation of the fitted ERG model is summarised as follows. The positive *2-star* parameter indicates that there is a tendency for multiple network partners. There is a significant difference in *t-value* of the *2-star* parameter between *high-cost* PCNs and *low-cost* PCNs (Table 5). A *t-test* in Table 6 shows this significance (t (10) = 2.13, p<0.05). The results show that on average, the *2-star* parameter for *low-cost* PCNs (M = 1.82, SE = 1.33) is less positive (in *t-values*) than the parameter for high-cost PCNs (M = 9.67, SE = 3.45). This indicates that the tendency for multiple network partners is more for *high-cost* PCNs than *low-cost* PCNs. It can be suggested from this trend that most of the actors of *high-cost* PCNs have multiple network partnerships with others. This means that *high-cost* PCNs are well connected, but there is a low probability of having any network-hub (i.e. a highly connected actor), which indicates that these networks (i.e. *high-cost* PCNs) are decentralised.

**Table 6:** ***t-test *for the *t-values *of different parameters of ERG model**

	**t-values of ERG model**					
**Parameter**	**Low readmission rate (mean)**	**High readmission rate (mean)**	**Low cost (mean)**	**High cost (mean)**	**t-test**	**P (T<=t) one-tail**
**est/sd(2-star)**			1.82	9.69	2.13	0.03
**est/std(AS)**	−1.73	−3.69			1.75	0.04
**est/std(A2P)**	−5.87	−21.58			3.04	0.01

Therefore, as PCNs become more centralised, the performance (i.e. inverse of cost) of the network will increase compared to decentralised PCNs.

There is a significant difference in *t-value* of the *alternating-k-star* parameter between *high- readmission* PCNs and *low-readmission* PCNs (Table 5). A *t-test* in Table 6 shows this significance (t (10) = 1.75, p<0.05). The results show that on average, the *k-star* parameter for *high-readmission* PCNs (M= −3.69, SE = 0.87) is more negative than the parameter for *low-readmission* PCNs (M = −1.73, SE = 0.71). The negative *alternating-k-star* parameter indicates that networks with some higher degree nodes are less probable, which means there is no actor playing the role of network-hubs. This means that *high-readmission* PCNs are more decentralised. This indicates that as PCNs become more centralised, the performance (i.e. inverse of readmission rate) of the network will improve compared to decentralised PCNs.

A significant difference in *t-value* for the *alternating-k-2-paths* parameter has been noticed between *high-readmission* PCNs and *low-readmission* PCNs (Table 5). A *t-test* in Table 6 shows this significance (t (10) =3.04, p<0.05). The results show that on average, the *alternating-k-two-paths* parameter for *high-readmission* PCNs (M = −21.58, SE = 4.55) is more negative than the *alternating-k-two-paths* for *low-readmission* PCNs (M = −5.87, SE = 2.47). The negative parameter of *alternating-k-two-path* indicates that the network does not tend to form cycles and this tendency is higher in *high-readmission* PCNs. So in *high-readmission* PCNs, the tendency to form cycles will be less.

## Discussion and conclusion

In this study, PCNs are constructed from the information of physicians’ visits to patients during their hospitalisation period. It is assumed that collaboration emerges between two physicians when they visit a common patient. It is a standard professional practice that when physicians visit patients they give advice or suggestions to patients based on their health condition and previous medication history deposited in the patient *log book*. All previous advice or suggestions prescribed by any physician to a patient have been taken into consideration during any subsequent physician visit to that patient. In addition, physicians often have been informed about the patient condition by other physicians who previously visited that patient. This kind of practice culture in healthcare organisations or hospitals establishes the validity and reliability of the construction process of PCN, and the generic nature of the research findings.

It is noticed that SNA measures of *density* for PCN has a positive correlation with *hospitalisation cost* and *readmission rate*. In a dense PCN, an increased number of links exists among physicians. Although connections with peers enable physicians a faster sharing of known knowledge [57], links with many peers significantly impacts an individual’s opportunity to share or create knowledge in a network (e.g. PCN). This is because when an individual has many links in a network, she needs to spend more time to maintain these relationships. Moreover, she will receive repetitive or contradictory knowledge, in addition to new knowledge, from many individuals which will make it difficult to summarise these shared knowledge [58]. Unlike *density*, the SNA measure of *betweenness centralisation* has negative correlation with both *hospitalisation cost* and *readmission rate*. From the perspective of a PCN structure, a high *betweenness centralisation* indicates that the structure of the corresponding PCN follows a *star-like* or *centralised* structure since *betweenness centralisation* reaches its highest value of 1 for a *star* network. A *star-like* or *centralised* network has few actors with higher *betweenness centrality* values. In this type of network, only a small number of actors play major collaboration and communication role. Therefore, in their corresponding hospitals, healthcare managers or administrators have to encourage or establish a *star-like* or *centralised* PCN in order to reduce both *hospitalisation cost* and *readmission rate*. A PCN with a flat network structure (i.e. members of that PCN have almost equal network participation) will have high *hospitalisation cost* and *readmission rate*.

Although this study finds that social network measures have statistically significant correlations with *hospitalisation cost* and *readmission rate* the corresponding correlation coefficient values do not show perfect correlations (i.e. a correlation coefficient value of 1) among them. The correlation coefficient values are ranging from 0.112 to 0.358 in absolute values. That means none of the relations shows perfect correlation. However, five of these values (see Table 2) are statistically significant at 0.01 and 0.05 levels (2-tailed). This is because of the sample size used in this study. This study uses 85 PCNs to explore the effects of different SNA measures on *hospitalisation cost* and *readmission rate*. A small correlation coefficient value could be statistically significant if sample size is high; whereas, for a small sample size a high correlation coefficient value would not be statistically significant [59]. A correlation coefficient value of 0.04, for instance, would be statistically significant for a sample size of 10,000 [59]. We also quantify the impact of social network measures on hospital outcome variables using simple linear regression models (see Table 3).

From the ERG model (i.e. *2-star*, *3-star*, *alternating-k-stars*, *alternating- k-triangles* and *alternating-k-two-paths* model), significant differences are noticed in *t-values* for different micro-structures between *high-readmission* PCNs and *low-readmission* PCNs. The *alternating-k-star* parameter shows a stronger negative value for *high-readmission* PCNs compared to *low-readmission* PCNs. Similarly, *alternating-k-two-path* shows more negative values for *high-readmission* PCNs compared to *low-readmission* PCNs. In summary, *high-readmission* PCNs are attributed with tronger negative values for *alternating-k-star* and *alternating-k-two-path* parameters. Negative *t-value* for an *alternating-k-star* parameter implies that networks with well-connected nodes are less probable (i.e. lack of the presence of network-hub). Negative *t-value* for an *alternating-k-two-path* parameter reveals that network actors are less likely to form cycles (i.e. networks are most sparse). From the interpretation of the findings of these two parameters (i.e. *alternating-k-star* and *alternating-k-two-path*) it can be concluded that *high-readmission* PCNs are decentralised. And *low-readmission* PCNs are more centralised compared to *high-readmission* PCNs. *Low-cost* PCNs and *high-cost* PCNs also have micro-structural differences that are statically significant. The *t-value* of *2-star* parameter is more positive for *high-cost* PCNs compared to *low-cost* PCNs. So, in *high-cost* PCNs, actors tend to have multiple partnerships with other network actors. That means *high-cost* PCNs are well connected and have a low chance of having any network-hub (i.e. highest degree actors). Therefore, *high-cost* PCNs are less centralised. And *low-cost* PCNs are more centralised compared to *high-cost* PCNs. To summarise, the findings from ERG models, both *low-readmission rate* and *low-cost* PCNs are more centralised compared to their counterparts. Therefore, in their corresponding PCNs, physicians have to be close to each other; and they should not work standalone or in disconnected small groups. A centralised PCN enables effective knowledge sharing among its member physicians, which eventually leads to better patient care [57].

In respect of the network data analysis using ERG model, this study intends to explore micro-level structures (e.g. 2-star and 3-star) that are associated with different PCNs characterised by the highest *hospitalisation cost* versus lowest *hospitalisation cost* and highest *readmission rate* versus lowest *readmission rate*. For this purpose, this study considers only the top 5 PCNs from all these four groups instead of considering all 85 PCNs of our research dataset.

This research is not without its limitations. First, we test relations between PCN attributes and healthcare care outcome measures and ERG models using the health insurance dataset only for THR patients. Thus, we need to consider dataset for other patients such as knee surgery patients or patients suffering from brain cancer in order to claim the general nature of the findings of this study. Second, we consider only quantitative measures (i.e. *hospitalisation cost* and *readmission rate*) as outcome variables. We do not consider any qualitative measures (e.g. patients’ satisfaction) as outcome variables. Finally, we consider only 20 PCNs for ERG modelling, which significantly limits the interpretation of the ERG findings. This is because, in this study we aim to explore structural differences in PCNs classified as high and low in terms of *hospitalisation cost* and *readmission rate*.

To conclude, this study first proposes a way to capture networks that evolve among physicians during the course of providing treatments to hospitalised patients. Second, SNA measures are utilised to explore PCNs. It is noticed that *density* has positive correlation with *hospitalisation cost* and *readmission rate*; whereas, *betweenness centralisation* is negatively associated with *hospitalisation cost* and *readmission rate*. *Degree centralisation* shows no significant correlation with *hospitalisation cost* and negative correlation with *readmission rate*. Finally, an ERG model is fitted with different types of PCNs (i.e. *low-cost* versus *high-cost* and *low-readmission* versus *high- readmission*). From the ERG model, it is found that PCNs, which are attributed with less negative *t-values* for *alternating-k-star* and *alternating-k-two-path* parameters, and lower *t-values* for *2-star* parameter, are more conducive to performance in terms of *low hospitalisation cost* and *low readmission rate* for patient hospital admissions. Healthcare managers and hospital administrators may follow the findings of this study in promoting the physician collaborations structure within their organisations.

## Endnote

^a^http://www.sna.unimelb.edu.au/pnet/pnet.html.

## Competing interests

The authors declare that they have no competing interests.

## Authors’ contributions

SU: study design, data collection, data analysis and writing. LH: study design, writing. JH: data analysis and writing. AA: writing. All authors read and approved the final manuscript.

## Pre-publication history

The pre-publication history for this paper can be accessed here:

http://www.biomedcentral.com/1472-6963/13/234/prepub

## References

[B1] Sawyer M Weeks K Goeschel CA Thompson DA Berenholtz SM Marsteller JA Lubomski LH Cosgrove SE Winters BD Murphy DJ Using evidence, rigorous measurement, and collaboration to eliminate central catheter-associated bloodstream infections Crit Care Med 2010 38 S292 20647786 10.1097/CCM.0b013e3181e6a165

[B2] Uddin S Hossain L Kelaher M Effect of physician collaboration network on hospitalization cost and readmission rate Eur J Public Health 2012 22 5 629 633 10.1093/eurpub/ckr153 22037593

[B3] Chukmaitov A Devers KJ Harless DW Menachemi N Brooks RG Strategy, structure, and patient quality outcomes in ambulatory surgery centers (1997–2004) Med Care Res Rev 2011 68 2 202 225 10.1177/1077558710378523 20829234

[B4] De Vreede GJ Briggs RO Collaboration engineering: designing repeatable processes for high-value collaborative tasks HICSS '05 Proceedings of the 38th Annual Hawaii International Conference on Systems Sciences 2005 17c

[B5] Knoben J Oerlemans L Proximity and inter-organizational collaboration: a literature review Int J Manag Rev 2006 8 2 71 89 10.1111/j.1468-2370.2006.00121.x

[B6] Cowan M Shapiro M Hays R Afifi A Vazirani S Ward C Ettner S The effect of a multidisciplinary hospitalist/physician and advanced practice nurse collaboration on hospital costs J Nurs Adm 2006 36 2 79 10.1097/00005110-200602000-00006 16528149

[B7] Tschannen D Kalisch B The effect of variations in nurse staffing on patient length of stay in the acute care setting West J Nurs Res 2009 31 2 153 18693216 10.1177/0193945908321701

[B8] Uddin S Hossain L Effects of physician collaboration network on hospital outcomes Australasian Workshop on Health Informatics and Knowledge Management 2012 Melbourne, Australia 67 73

[B9] Knaus WA Draper EA Wagner DP Zimmerman JE An evaluation of outcome from intensive care in major medical centers Annals of Internal Medicine 1986 104 3 410 10.7326/0003-4819-104-3-410 3946981

[B10] Baggs J Schmitt M Mushlin A Mitchell P Eldredge D Oakes D Hutson A Association between nurse-physician collaboration and patient outcomes in three intensive care units Crit Care Med 1999 27 9 1991 10.1097/00003246-199909000-00045 10507630

[B11] Lindeke L Sieckert A Nurse-physician workplace collaboration Online J Issues Nurs 2005 10 1 10 15727548

[B12] Uddin MS Hossain L Exploring physical, mental and psychological health for elders through their personal networks In e-Health Networking, Applications and Services 2009 Sydney: IEEE 29 35

[B13] Wasserman S Faust K Social network analysis: Methods and applications 2003 Cambridge: Cambridge University Press

[B14] Carrington P Scott J Wasserman S Models and methods in social network analysis 2005 Cambridge: Cambridge Univ Pr

[B15] Snijders TAB Pattison PE Robins GL Handcock MS New specifications for exponential random graph models Sociol Methodol 2006 36 1 99 153 10.1111/j.1467-9531.2006.00176.x

[B16] Robins G Pattison P Woolcock J Missing data in networks: exponential random graph (P*) models for networks with non-respondents Soc Networks 2004 26 3 257 283 10.1016/j.socnet.2004.05.001

[B17] Saul ZM Filkov V Exploring biological network structure using exponential random graph models Bioinformatics 2007 23 19 2604 2611 10.1093/bioinformatics/btm370 17644557

[B18] Hamra J Uddin S Hossain L Exponential random graph modeling of communication networks to understand organizational crisis SIGMIS annual conference on Computer personnel research: 2011 2011 78: ACM 71

[B19] Chen LM Jha AK Guterman S Ridgway AB Orav EJ Epstein AM Hospital cost of care, quality of care, and readmission rates: penny wise and pound foolish? Arch Intern Med 2010 170 4 340 20177036 10.1001/archinternmed.2009.511

[B20] Ross JS Chen J Lin Z Bueno H Curtis JP Keenan PS Normand SLT Schreiner G Spertus JA Vidán MT Recent national trends in readmission rates after heart failure hospitalization Circ Heart Fail 2010 3 1 97 10.1161/CIRCHEARTFAILURE.109.885210 19903931 PMC2830811

[B21] Reiter KL Sandoval GA Brown AD Pink GH CEO compensation and hospital financial performance Med Care Res Rev 2009 66 6 725 738 10.1177/1077558709338479 19605619 PMC3014258

[B22] Mahmoud N Turpin R Yang G Saunders W Impact of surgical site infections on length of stay and costs in selected colorectal procedures Surg Infect 2009 10 6 539 10.1089/sur.2009.006 19708769

[B23] Husted H Holm G Jacobsen S Predictors of length of stay and patient satisfaction after hip and knee replacement surgery: fast-track experience in 712 patients Acta Orthop 2008 79 2 168 173 10.1080/17453670710014941 18484241

[B24] Hunt JS Siemienczuk J Pape G Rozenfeld Y MacKay J LeBlanc BH Touchette D A randomized controlled trial of team-based care: impact of physician-pharmacist collaboration on uncontrolled hypertension J Gen Intern Med 2008 23 12 1966 1972 10.1007/s11606-008-0791-x 18815843 PMC2596500

[B25] Arbuthnott A Sharpe D The effect of physician-patient collaboration on patient adherence in non-psychiatric medicine Patient Educ Couns 2009 77 1 60 67 10.1016/j.pec.2009.03.022 19395222

[B26] Burns LR Muller RW Hospital physician collaboration: landscape of economic integration and impact on clinical integration Milbank Q 2008 86 3 375 434 10.1111/j.1468-0009.2008.00527.x 18798884 PMC2690342

[B27] Gaboury I Bujold M Boon H Moher D Interprofessional collaboration within Canadian integrative healthcare clinics: Key components Soc Sci Med 2009 69 5 707 715 10.1016/j.socscimed.2009.05.048 19608320

[B28] Cunningham FC Ranmuthugala G Plumb J Georgiou A Westbrook JI Braithwaite J Health professional networks as a vector for improving healthcare quality and safety: a systematic review BMJ Quality & Safety 2012 21 3 239 249 10.1136/bmjqs-2011-000187 22129933 PMC3285140

[B29] Sommers L Marton K Barbaccia J Randolph J Physician, nurse, and social worker collaboration in primary care for chronically ill seniors Arch Intern Med 2000 160 12 1825 1833 10.1001/archinte.160.12.1825 10871977

[B30] Netting F Williams F Case manager-physician collaboration: implications for professional identity, roles, and relationships Health Soc Work 1996 21 3 (10.1093/hsw/21.3.216 8854126

[B31] Fattore G Frosini F Salvatore D Tozzi V Social network analysis in primary care: the impact of interactions on prescribing behaviour Health Policy 2009 92 2 141 148 19356822 10.1016/j.healthpol.2009.03.005

[B32] Meltzer D Chung J Khalili P Marlow E Arora V Schumock G Burt R Exploring the use of social network methods in designing healthcare quality improvement teams Soc Sci Med 2010 71 6 1119 1130 10.1016/j.socscimed.2010.05.012 20674116 PMC2931366

[B33] Fagin CM Collaboration between nurses and physicians: no longer a choice Acad Med: journal of the Association of American Medical Colleges 1992 67 5 295 10.1097/00001888-199205000-00002 1575859

[B34] Baggs JG Schmitt MH Collaboration between nurses and physicians J Nurs Scholarsh 1988 20 3 145 149 10.1111/j.1547-5069.1988.tb00055.x 3049311

[B35] Christensen C Larson JR Collaborative medical decision making Med Decis Making 1993 13 4 339 346 10.1177/0272989X9301300410 8246706

[B36] Lindeke LL Sieckert AM Nurse-physician workplace collaboration Online J Issues Nurs 2005 10 1 5 15727548

[B37] Kramer M Schmalenberg C Securing" good" nurse/physician relationships Nurs Manage 2003 34 7 34 10.1097/00006247-200307000-00013 12843717

[B38] Atkinson P Medical talk and medical work: the liturgy of the clinic 1995 London: Sage Publications Ltd

[B39] Bosk CL Forgive and remember: managing medical failure 1979 Chicago: University of Chicago Press

[B40] Freidson E Doctoring together: A study of professional social contro 1980 Chicago: University of Chicago Press

[B41] Millman M The unkindest cut: Life in the backrooms of medicine 1977 Morrow Quill Paperbacks

[B42] Pettinari CJ Task, talk and text in the operating room: study in medical discourse (Advances in discourse processes) (v. 33) Recherche 1989 67 02 10.1111/1467-9566.ep11375292 29363804

[B43] Atkinson P The ethnography of a medical setting: reading, writing, and rhetoric Qual Health Res 1992 2 4 451 474 10.1177/104973239200200406

[B44] Hunter KM Doctors' stories: The narrative structure of medical knowledge 1993 Princeton Univ Pr

[B45] Bavelas A Communication patterns in task-oriented groups J Acoust Soc Am 1950 22 725 730 10.1121/1.1906679

[B46] Freeman L Centrality in social networks: conceptual clarification Soc Networks 1978 1 3 215 239 10.1016/0378-8733(78)90021-7

[B47] Freeman L Roeder D Mulholland R Centrality in social networks: II. experimental results Soc Networks 1979 2 80 119 141

[B48] Wasserman S Pattison P Logit models and logistic regressions for social networks: I. An introduction to Markov graphs and P Psychometrika 1996 61 3 401 425 10.1007/BF02294547

[B49] Uddin S Hamra J Hossain L Exploring communication networks to understand organizational crisis using exponential random graph models Comput Math Organ Theory 2013 19 1 25 41 10.1007/s10588-011-9104-8

[B50] Robins G Snijders T Wang P Handcock M Pattison P Recent developments in exponential random graph (p*) models for social networks Soc Networks 2007 29 2 192 215 10.1016/j.socnet.2006.08.003

[B51] Frank O Strauss D Markov graphs 1986 Alexandria, VA, ETATS-UNIS: American Statistical Association

[B52] Robins G Pattison P Kalish Y Lusher D An introduction to exponential random graph (p*) models for social networks Soc Networks 2007 29 2 173 191 10.1016/j.socnet.2006.08.002

[B53] Strauss D Ikeda M Pseudolikelihood estimation for social networks J Am Stat Assoc 1990 85 409 204 212 10.1080/01621459.1990.10475327

[B54] Carley K Center for Computational Analysis of Social and Organizational Systems (CASOS), Institute for Software Research International (ISRI) 2010 5000 Forbes Avenue Pittsburgh, PA 15213–3890: School of Computer Science, Carnegie Mellon University

[B55] Baron RM Kenny DA The moderator-mediator variable distinction in social psychological research: conceptual, strategic, and statistical considerations J Pers Soc Psychol 1986 51 6 1173 1182 3806354 10.1037//0022-3514.51.6.1173

[B56] Snijders TAB Van de Bunt GG Steglich CEG Introduction to stochastic actor-based models for network dynamics Soc Networks 2010 32 1 44 60 10.1016/j.socnet.2009.02.004

[B57] Ryu S Ho S Han I Knowledge sharing behavior of physicians in hospitals Expert Syst Appl 2003 25 1 113 122 10.1016/S0957-4174(03)00011-3

[B58] Burt R Structural holes: The social structure of competition 1992 Cambridge, Massachusetts: Harvard Univ Pr

[B59] Field A Discovering statistics using SPSS 2009 London: Sage Publications Ltd

